# Protective Effects of Selol Against Sodium Nitroprusside-Induced Cell Death and Oxidative Stress in PC12 Cells

**DOI:** 10.1007/s11064-016-2046-2

**Published:** 2016-09-02

**Authors:** Agnieszka Dominiak, Anna Wilkaniec, Piotr Wroczyński, Henryk Jęśko, Agata Adamczyk

**Affiliations:** 1 Department of Bioanalysis and Drug Analysis, Medical University of Warsaw, 1 Banacha St., 02-097 Warsaw, Poland; 2 Department of Cellular Signalling, Mossakowski Medical Research Centre Polish Academy of Sciences, 5 Pawińskiego St., 02-106 Warsaw, Poland

**Keywords:** Antioxidative defense, Cytoprotection, Oxidative/nitrosative stress, Organic selenium compound, Selenoenzymes, Selol

## Abstract

Selol is an organic selenitetriglyceride formulation containing selenium at +4 oxidation level that can be effectively incorporated into catalytic sites of of Se-dependent antioxidants. In the present study, the potential antioxidative and cytoprotective effects of Selol against sodium nitroprusside (SNP)-evoked oxidative/nitrosative stress were investigated in PC12 cells and the underlying mechanisms analyzed. Spectrophoto- and spectrofluorimetic methods as well as fluorescence microscopy were used in this study; mRNA expression was quantified by real-time PCR. Selol dose-dependently improved the survival and decreased the percentage of apoptosis in PC12 cells exposed to SNP. To determine the mechanism of this protective action, the effect of Selol on free radical generation and on antioxidative potential was evaluated. Selol offered significant protection against the elevation of reactive oxidative species (ROS) evoked by SNP. Moreover, this compound restored glutathione homeostasis by ameliorating the SNP-evoked disturbance of GSH/GSSG ratio. The protective effect exerted by Selol was associated with the prevention of SNP-mediated down-regulation of antioxidative enzymes: glutathione peroxidase (Se-GPx), glutathione reductase (GR), and thioredoxin reductase (TrxR). Finally, GPx inhibition significantly abolished the cytoprotective effect of Selol. In conclusion, these results suggest that Selol effectively protected PC12 cells against SNP-induced oxidative damage and death by adjusting free radical levels and antioxidant system, and suppressing apoptosis. Selol could be successfully used in the treatments of diseases that involve oxidative stress and resulting apoptosis.

## Introduction

Selenium (Se) is an essential trace element that is biologically active in the form of Se-containing selenoproteins. This mineral when replaced the sulfur atom in the amino acid cysteine (Cys) forms selenocysteine (Sec), which is the 21st proteinogenic amino acid. Selenium appears to have a multifaceted role in the homoeostasis of central nervous system (CNS) including the maintenance of cellular redox status, mitochondrial dynamics, regulation of Ca^2+^ channels, and modulation of neurogenesis. Oxidative stress is defined as an imbalance between the generation and detoxification of reactive oxygen species (ROS). Brain is particularly vulnerable to oxidative stress due to its high oxygen demand (it takes up to 20 % of the total oxygen consumed), high levels of iron and unsaturated fatty acids along with relatively inefficient antioxidative defense [1]. Oxidative stress plays an important role in brain aging as well as in various neurodegenerative diseases [2, 3] along with the deregulation of nitric oxide (NO)-based signaling, neurotransmission, and immune function [4]. The association between the risk of neurodegenerative diseases and the antioxidative capacity has been demonstrated by numerous studies, suggesting the importance of antioxidants as disease-preventing agents [5–7]. Free radical-dependent macromolecular damage is involved in the pathogenesis of Alzheimer’s (AD) and Parkinson’s (PD) diseases, multiple sclerosis (MS), amyotrophic lateral sclerosis (ALS), and other age-related neurodegenerative disorders [4, 8–12]. Various experimental tools have been developed to stimulate oxidative/nitrosative stress in biological material. Sodium nitroprusside (SNP) releases NO, but can also elevate cellular levels of Fe^2+^, H_2_O_2_, and [Fe(CN_6_)]^4−^ [13]. Direct antioxidant role of Se in the CNS seems to be well established. Se is an integral constituent of antioxidant enzymes, such as glutathione peroxidases (GPxs) and thioredoxin reductases (TrxRs). Other selenoproteins cleave iodine–carbon bonds in the metabolism of thyroid hormones (iodothyronine deiodinase; DIOs) and are involved in the regulation of Ca^2+^ influx (selenoprotein M). Yet others catalyze the reduction of protein-based methionine-R-sulfoxide to methionine (selenoprotein R) or chelate heavy metals (selenoprotein P) [14–16]. Accumulation of free radicals and generation of excessive ROS in aged brain are accompanied with lower Se levels, which might significantly contribute to neuropsychological decline [17]. Se deficit is further escalated (along ROS accumulation) in neurodegenerative disorders, particularly in the brains of AD patients [18–22]. Se concentration tends to decrease also in the serum of patients with MS [23] and in autopsy brains from patients with Huntington’s disease [24].

The association of oxidative stress and selenium disturbances with neurodegenerative disorders suggests that Se administration might be useful in their prevention and treatment. However, the currently available evidence on the precise effects of different forms and doses of Se is inconclusive. Therefore, there is still a need for novel, more effective compounds. Efficient uptake and metabolism of dietary Se strongly depend on its chemical form. Water-soluble selenite and selenate are commonly used inorganic forms of Se [25]. However, the results of Letavayová et al. suggest that inorganic Se donors might be more toxic and have lower intestinal absorption efficacy than organic Se species [26]. Organic Se sources mostly include the following amino acids: SeMet (**se**leno**met**hionine), Sec (**se**leno**c**ysteine), and MeSeCys (**me**thyl**se**leno**cys**teine) [27]. Other organic Se compounds include selenoneine, Se-enriched yeast, and synthetic ethaselen or ebselen [28, 29].

Drawing from the currently available data, we set out to investigate the organic Se-containing compound, Selol, which was first synthesized at Warsaw Medical University, Poland (Polish Patent 1999) [30]. Selol is a semi-synthetic mixture of organic selenitetriglycerides obtained from sunflower oil, containing Se at the +4 oxidation level. Previous results indicated that Selol did not exhibit any toxic potential after parenteral administration at concentrations of 500 mg/kg *s.c*. and 100 mg/kg *i.p*. and below, and also it has not revealed any mutagenic potential up to 5000 Se µg/plate in *Salmonella typhimurium*/microsome mutagenicity assay [31, 32]. The compound undergoes rapid resorption from the digestive system and it is widely distributed in the organism. In particular, this lipophilic compound has the ability to cross the blood–brain barrier. Furthermore, it is completely eliminated from the organism after 24 h from administration, avoiding accumulation and toxic effects [33]. While the efficiency of other organic selenium compounds against oxidative stress has been highlighted, it remains unknown whether Selol can antagonize SNP-induced damage.

The aim of the present study was to investigate the potential antioxidative and cytoprotective effects of Selol against SNP-evoked oxidative/nitrosative stress in rat pheochromocytoma (PC12) dopaminergic cells and to explain the underlying mechanism of these effects.

## Materials and Methods

### Compounds and Reagents

Selol was synthesized at the Department of Bioanalysis and Drug Analysis at Medical University of Warsaw (Polish Patent 1999). A micellar solution of Selol was prepared *ex tempore* (based on lecithin, water, and Selol), with a declared selenium concentration of 5 % (w/v).

Dulbecco’s modified Eagle’s medium (DMEM), fetal bovine serum (FBS), horse serum (HS), penicillin, streptomycin, l-glutamine, 3-(4,5-dimethyl-2-tiazolilo)-2,5-diphenyl-2*H*-tetrazolium bromide (MTT), 2′-(4-hydroxyphenyl)-5-(4-methyl-1-piperazinyl)-2,5′-bi-1*H-*benzimidazoletrihydrochloridehydrate (Hoechst 33258), TRI-reagent, polyethylenoimine (PEI), dimethyl sulfoxide (DMSO), sodium aurothiomalate (SAu), sodium selenite (Na_2_SeO_3_), reduced glutathione (GSH), glutathione reductase (GR), the reduced form of nicotinamide adenine dinucleotide phosphate (NADPH), sodium azide, tert-butylperoxide, 2-vinylpyridine, triethanolamine, metaphosphoric acid, bovine serum albumin (BSA), and all other common reagents were purchased from Sigma-Aldrich (St. Louis, MO, USA). Reagents for reverse transcription (High Capacity RNA-to-cDNA Master Mix) and PCR (Gene Expression Master Mix) were from Applied Biosystems (Applied Biosystems, Foster City, CA, USA).

### Cell Culture

The studies were carried out using rat pheochromocytoma cells (PC12) that were a kind gift from Prof. A. Eckert (University of Basel, Basel, Switzerland). All cell lines were cultured in DMEM supplemented with 10 % heat-inactivated FBS and 5 % heat-inactivated HS, 50 U/ml penicillin/streptomycin, and 2 mM l-glutamine. Cells were maintained at 37 °C in a humidified incubator containing 5 % CO_2_ atmosphere.

### Cell Treatment Protocols

Equal PC12 cell numbers were seeded into 96-well 0.1 % polyethyleneimine-coated plates at density of 7.5 × 10^4^/ml for MTT test, 2 × 10^5^/ml for determination of apoptosis as well as the intracellular ROS levels and were grown on 100 mm^2^ dishes to 90 % confluence for enzyme activity assays or on 60 mm^2^ dishes to 90 % confluence for enzyme expression. After 24 h, the growth medium was changed to a low-serum medium (DMEM supplemented with 2 % FBS, 50 U/ml penicillin/streptomycin, and 2 mM l-glutamine). Then the PC12 cells were treated with Selol or sodium selenite, SNP (0–1 mM), and GPx inhibitor (sodium aurothiomalate, SAu, 2 µM) for 24 h.

### Determination of Cell Survival Using MTT Test

Cellular viability was evaluated by the reduction of 2-(4,5-dimethylthiazol-2-yl)-2,5-diphenyltetrazolium bromide (MTT) to formazan. After 24 h of treatment with the tested compounds, MTT (2.5 mg/ml) was added to all of the wells and was incubated at 37 °C for 2 h. Then the medium was removed, the formazan crystals were dissolved in DMSO, and absorbance at 595 nm was measured. The results were presented as percent of control.

### Determination of Apoptosis

The formation of apoptotic bodies was determined by microscopic analysis of the cells stained with Hoechst 33258. Shortly, after 8 h of incubation in the presence of tested compounds, the cells were fixed, stained, and examined under a fluorescence microscope (Olympus BX51, Olympus Corp., Tokyo, Japan) and photographed with a digital camera (Olympus DP70). Cells from ten random fields were counted under a 40× objective and the percentage of typical apoptotic nuclear morphology (nuclear shrinkage, condensation) was calculated.

### Determination of Se-Dependent Glutathione Peroxidase Activity

After 24 h of treatment with the tested compounds, the glutathione peroxidase activity was determined by spectrophotometric assay based on the method of Paglia and Valentine [34], modified by Wendel [35]. Cells were homogenized in cold buffer (50 mM Tris-HCl, pH = 7.5, 5 mM EDTA, 1 mM DTT) and centrifuged (10,000×*g*, 15 min, 4 °C), and the supernatant (20 µl) was used to analyze enzyme activity and to determine protein concentration. Se-glutathione peroxidase (Se-GPx) activity was measured indirectly with *tert*-butyl hydroperoxide (*t*-Bu-OOH) as a substrate in a reaction that progresses proportionally to the rate of NADPH oxidation by GR. The reaction mixture contained optimized concentrations of the following chemicals in final volume 220 µl: reduced glutathione (1.0 mM), NADPH (65 µM), and sodium azide (0.17 mM). The reaction was started by adding *t*-butyl hydroperoxide (0.02 mM) and carried out at 25 °C. The decrease in absorbance (proportional to Se-GPx activity) was measured once every 5 min at 340 nm. The results were expressed as U/mg protein.

### Determination of Glutathione Reductase Activity

After 24 h of treatment with the tested compounds, GR activity was measured using spectrophotometric GR Assay Kit (Item No. 703202, Cayman Chemical, Ann Arbor, USA). Cells were homogenized in cold buffer (50 mM potassium phosphate, pH 7.5, containing 1 mM EDTA) and centrifuged (10,000×*g*, 15 min, 4 °C), and the resulting supernatant (20 µl) was used to analyze enzyme activity and to determine protein concentration. GR catalyzes the reduction of oxidized glutathione (GSSG) to GSH with a consumption of one NADPH. Briefly, the reaction mixture contained 50 mM potassium phosphate (pH 7.5), 1 mM EDTA and 1 mM GSSG. The reaction was initiated by adding 50 µl NADPH and the decrease in absorbance caused by the oxidation of NADPH to NADP^+^ was measured once every 5 min at 340 nm. The results were presented as nmol/min/mg of protein.

### Determination of Glutathione Levels

After 24 h of treatment with the tested compounds, total (i.e., both oxidized and reduced) and oxidized glutathione (GSSG) levels were measured using enzymatic Glutathione Assay Kit (Item No. 703002, Cayman Chemical, Ann Arbor, USA). Cells were homogenized in cold buffer (50 mM MES, pH 6–7, containing 1 mM EDTA) and centrifuged (10,000×*g*, 15 min, 4 °C). The supernatant was used to determine protein content and is deproteinated before analysis. GSSG concentration was determined by derivatization technique. The reaction was initiated by adding freshly prepared assay cocktail and the change in absorbance was detected at 405 nm after 25 min. GSH was quantified using the following equation: $\text{GSH}=\text{Total}\ \text{glutathione}-2\times \text{GSSG}$. The results were presented as nmol/mg of protein.

### Determination of Thioredoxin Reductase Activity

After 24 h of treatment with the tested compounds, thioredoxin reductase activity was measured by Thioredoxin Reductase Colorimetric Assay Kit (Item No. 10007892, Cayman Chemical, Ann Arbor, USA). Cells were homogenized in cold buffer (50 mM potassium phosphate, pH 7.4, containing 1 mM EDTA) and centrifuged (10,000×*g*, 15 min, 4 °C). The supernatant (20 µl) was used to analyze enzyme activity and to determine protein content. The reaction was initiated by adding 5,5-dithiobis-(2nitrobenzoic acid) (DTNB) solution and NADPH and the change in absorbance caused by the formation of 2-nitro-5thiobenzoic acid (TNB) was measured once every 5 min at 405 nm. Measurement of TrxR activity by DTNB reduction in the absence and in the presence of aurothiomalate (20 µM), allows for correction of non-thioredoxin reductase-independent DTNB reduction. Briefly, the reaction mixture contained 50 mM potassium phosphate (pH = 7.0), 50 mM potassium chloride, 1 mM EDTA, and 0.2 mg/ml BSA. The results were presented as µmol/min/mg of protein.

### Determination of the Intracellular Levels of Reactive Oxygen Species

The intracellular ROS level was measured in adherent cells in 96-well black plates for 8 h as described previously [36]. The assay is based on the conversion of 2′,7′-dichlorodihydrofluorescein diacetate (H2DCF-DA) to 2′,7′-dichlorodihydrofluorescin (H2DCF), which is then oxidized by free radicals to 2′,7′-dichlorofluorescein (DCF). Fluorescence was measured at 37 °C in Omega spectrofluorimeter (BMG Labtech GMBH, Ortenberg, Germany) at excitation and emission wavelengths of 488 and 525 nm, respectively. The results were presented as fluorescence intensity, percent of control.

### Determination of Reactive Oxygen Species in Cell-Free System

The assay is based on the alkaline hydrolysis of 2′,7′-dichlorodihydrofluorescein diacetate (H_2_DCF-DA) to 2′,7′dichlorodihydrofluorescin (H_2_DCF). Methanol, 2.5 mM 2′,7′-dichlorodihydrofluorescein diacetate (H_2_DCF-DA), and 2 M KOH were mixed in a ratio of 1:1:0.5 and this mixture was kept in darkness at room temperature for 1 h. Then, HCl was added to neutralize the mixture (pH = 7). H_2_DCF is oxidized by free radicals to 2′,7′-dichlorofluorescein (DCF). Fluorescence was measured for 70 min in Omega spectrofluorimeter (BMG Labtech GMBH, Ortenberg, Germany) at excitation and emission wavelengths of 488 and 525 nm, respectively. The results were presented as fluorescence intensity.

### Quantitative Real-Time PCR

Reverse transcription was performed using High Capacity cDNA Reverse Transcription Kit according to the manufacturer’s protocol (Applied Biosystems, Foster City, CA, USA). After 24 h of treatment with the tested compounds, the mRNA levels for selected genes were analyzed using TaqMan Gene Expression Assays (Applied Biosystems) according to the manufacturer’s instructions. Actb was used in all studies as the reference gene. Plates were analyzed on ABI PRISM 7500 apparatus (Applied Biosystems). The relative levels of mRNA (Rq) were calculated using the ∆∆Ct method. The results were presented as percent of control.

### Protein Determination

Protein content was determined using Bradford protein assay according to the manufacturer’s protocol [37]. Standard curve for the assays was prepared with BSA. The absorbance of protein-bound dye [Coomassie Brilliant Blue G-250 (CBB G-250)] was measured at 595 nm.

### Statistical Analysis

The results were expressed as mean values ± S.E.M. Differences between means were analyzed using one-way ANOVA followed by the Newman–Keuls post hoc tests and p < 0.05 was considered statistically significant. The statistical analyzes were performed by using Graph Pad Prism version 5.0 (Graph Pad Software, San Diego, CA).

## Results

**Fig. 1:**
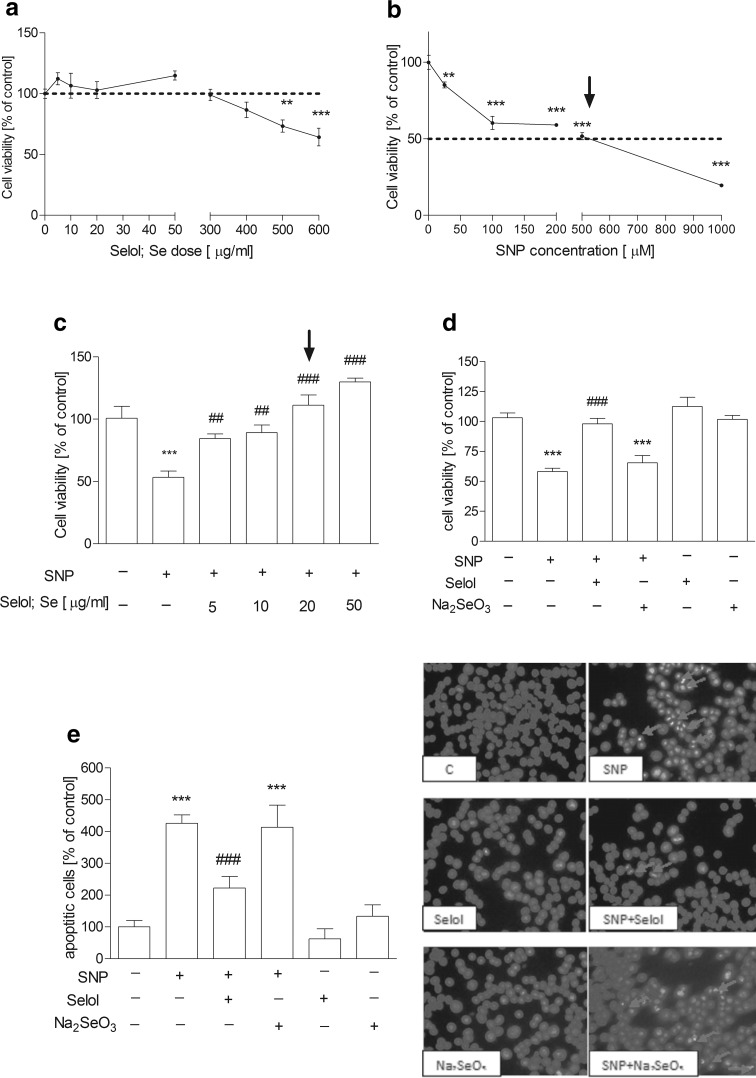
The effect of Selol on SNP-evoked cell damage and death. **a** Concentration-dependent effect of Selol at selenium doses 5–600 µg/ml on the viability of PC12 cells after 24 h of exposure. **b** The effect of SNP (0.025–1 mM) on PC12 cell viability. **c** The dose-dependent effect of Selol (5–50 μg/ml Se) on SNP-induced PC12 cell death. **d** Selol at the selected dose of 20 μg/ml Se prevented the reduction of cell viability evoked by 0.5 mM SNP while sodium selenite (Na_2_SeO_3_, 20 μg Se/ml) had no influence. **e** After 8 h of incubation, the level of apoptotic bodies in SNP- and Selol**-**/sodium selenite-treated cells. In the *right panel*, representative fluorescent photomicrographs are shown; *arrows* indicate apoptotic bodies. Data expressed as percentage of apoptotic cells, n = 4–10. **p < 0.01; ***p < 0.001 versus control (nontreated) cells; ^##^p < 0.01; ^###^p < 0.001 versus SNP

**Fig. 2:**
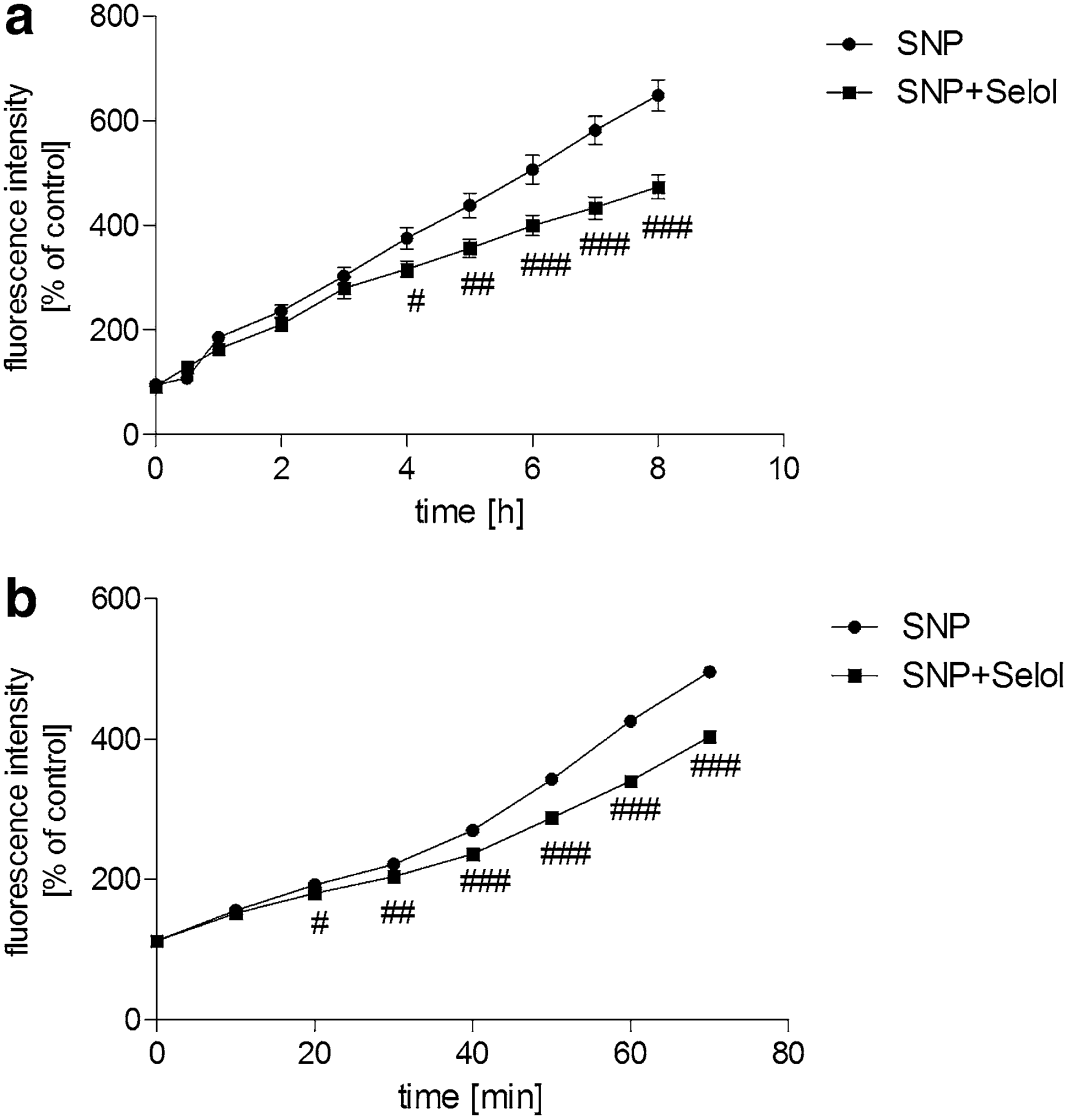
The effect of Selol on time-dependent ROS generation induced by SNP. **a** PC12 cells treated with Selol (20 μg Se/ml) and SNP (0.5 mM) stained by DCFH-DA were subjected to fluorimetric analysis for 8 h. **b** Fluorescence intensity was measured in the presence of Selol (20 μg Se/ml) and SNP (0.5 mM) for 70 min in cell-free system. Fluorescence intensity is expressed as percentage of control; n = 6–8; ^#^p < 0.05; ^##^p < 0.01; ^###^p < 0.001 versus SNP

**Fig. 3:**
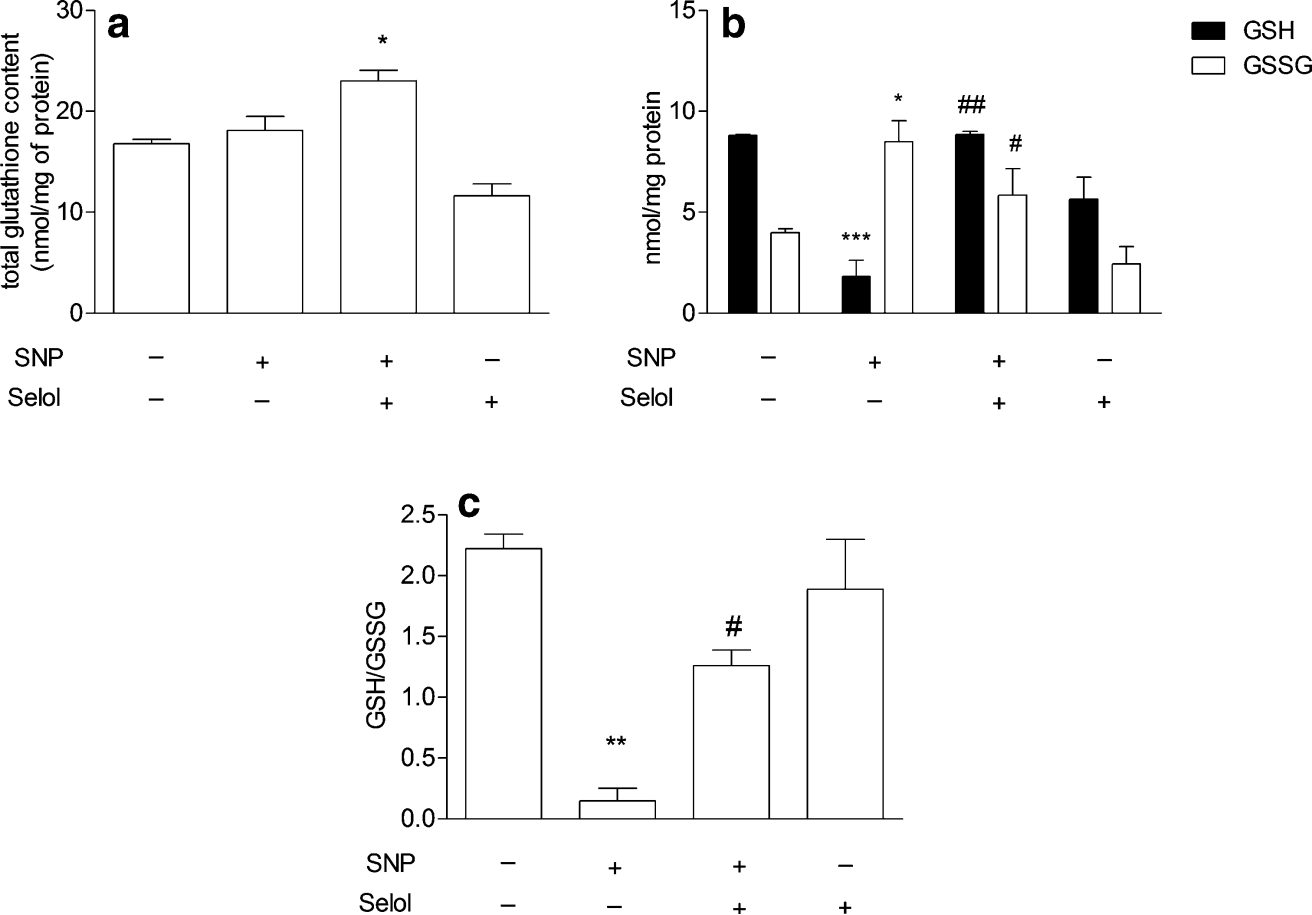
The effect of Selol on SNP-mediated glutathione redox imbalance. **a** Total glutathione and **b** oxidized (GSSG) as well as reduced (GSH) glutathione level after 24 h of incubation with Selol (20 μg/ml Se) and SNP (0.5 mM). **c** GSH/GSSG ratio. n = 4–6; *p < 0.05; **p < 0.01;***p < 0.001 versus control; ^#^p < 0.05; ^##^p < 0.01 versus SNP

**Fig. 4:**
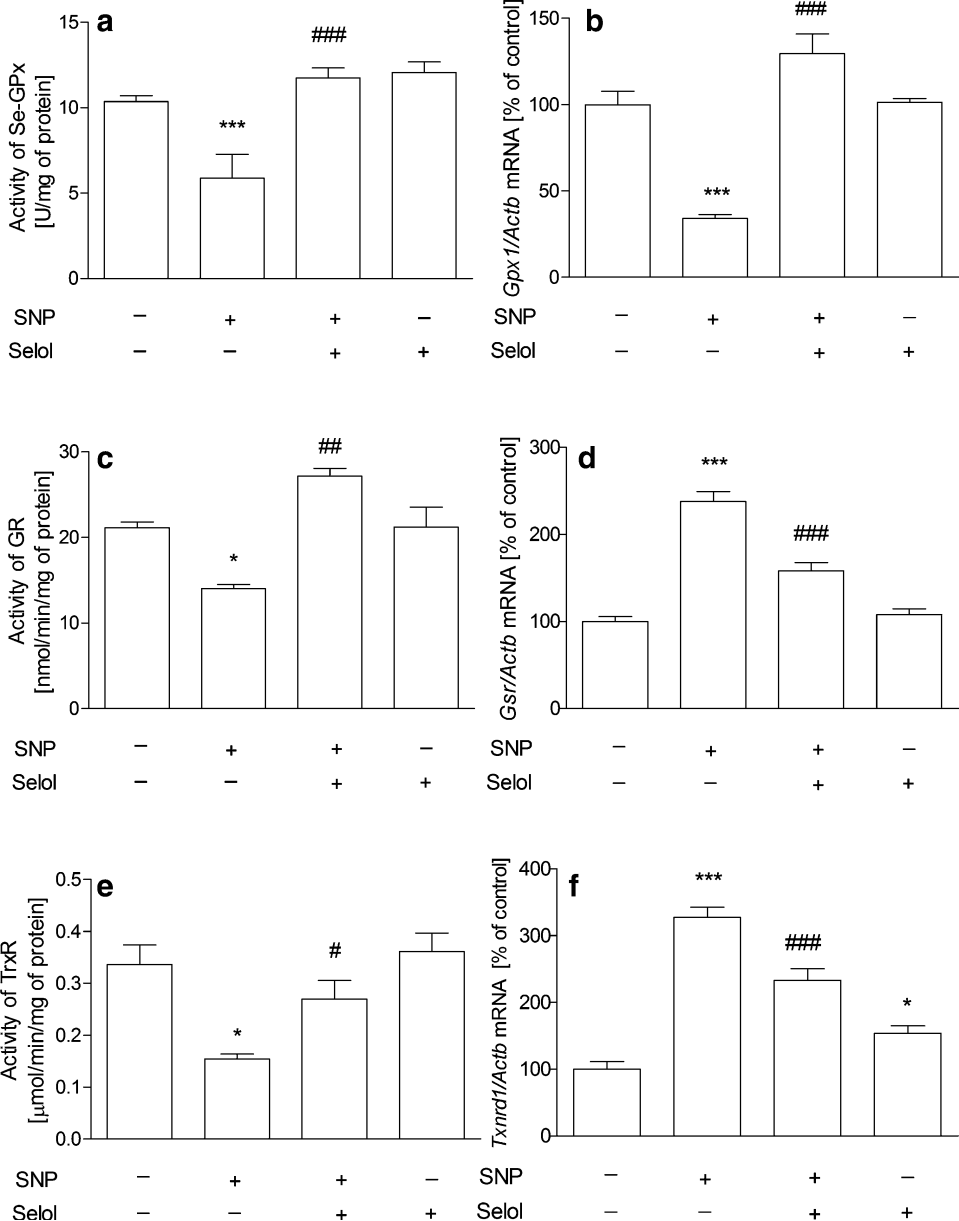
The effect of Selol on SNP-evoked antioxidant enzyme changes. The activities and mRNA expression of antioxidant enzymes after 24 h of incubation of PC12 cells with Selol (20 μg Se/ml) and SNP (0.5 mM) were determined spectrophotometrically and by real-time quantitative RT-PCR as described in section “Materials and Methods”. **a, b** Se-GPx; **c, d** GR and **e, f** TrxR. n = 4–10; *p < 0.05; ***p < 0.001 versus control; ^#^p < 0.05; ^##^p < 0.01; and ^###^p < 0.001 versus SNP

**Fig. 5:**
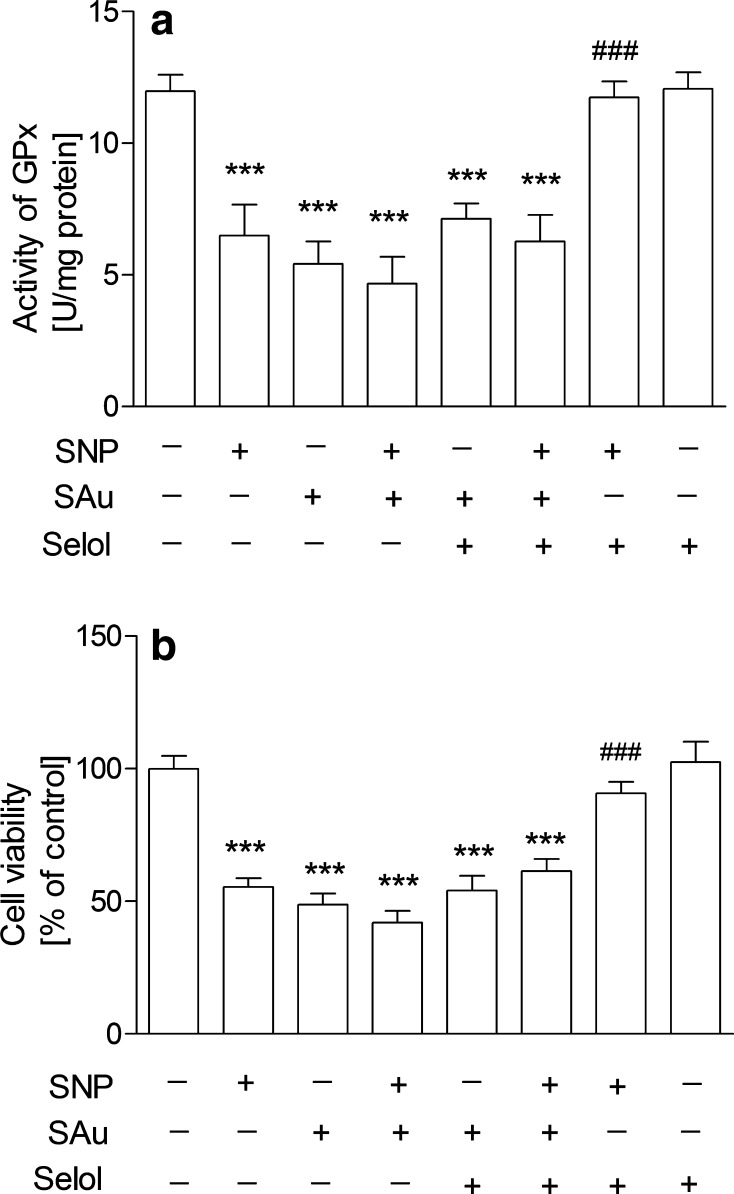
Sodium aurothiomalate (SAu) abolished the protective effect of Selol against SNP toxicity. PC12 cells were exposed to Selol at 20 μg/ml Se, SAu (2 μM), and SNP (0.5 mM) for 24 h, and then the enzyme activity and cell viability were analyzed using spectrophotometry. **a** The effect of SAu on GPx activity. **b** MTT test of cell survival. n = 4–8; ***p < 0.001 versus control; ^###^p < 0.001 versus SNP

The study was started from the evaluation of the effects of Selol and SNP on PC12 cell viability. The MTT [3-(4,5-dimethylthiazol-2-yl)-2,5-diphenyltetrazolium bromide] tetrazolium reduction assay revealed that Selol containing selenium at 5–400 μg/ml did not affect cell survival (Fig. 1a). Only at doses 500 µg/ml or higher, Se reduced cell viability (Fig. 1a). SNP (25–1000 μM, Fig. 1b) significantly decreased cell viability in a dose-dependent manner. SNP at 500 µM was selected for further studies based on the cell viability score 50 (CVS50, concentration at which viability was ≤50 % of control) as indicated by the arrow in the figure. To select appropriate Se dose for cytoprotection against SNP-evoked toxicity, the dose-dependent effect of Selol (Se concentration, 5–50 μg/ml) on SNP-induced PC12 cell death was evaluated (Fig. 1c). Selol containing Se at a dose 20 μg/ml completely inhibited SNP-evoked reduction of cell viability (Fig. 1c). Therefore, Selol containing 20 μg Se/ml was chosen as the minimum effective dose to be used in the subsequent experiments as indicated by the arrow in the figure. The cytoprotective effect of Selol was compared with the influence of inorganic Se compound, sodium selenite (Na_2_SeO_3_). As shown in Fig. 1d, Na_2_SeO_3_ had no protective effect against cell damage evoked by SNP. Quantitative analysis of apoptosis was carried out via microscopic examination of cell nuclei stained with DNA-binding fluorochrome Hoechst 33258. Our result indicated that SNP induced apoptosis in PC12 cells with fragmentation of nuclei and formation of apoptotic bodies. Selol treatment significantly decreased the percentage of apoptotic PC12 cells after SNP treatment (Fig. 1e), while Na_2_SeO_3_ was unable to protect against SNP-evoked apoptosis (Fig. 1e). In order to determine if Selol might influence the oxidative/nitrosative stress evoked by SNP, we evaluated the effect of Selol on SNP-induced ROS accumulation. As shown in Fig. 2a, cells exposed to SNP exhibited higher oxidative stress, evaluated by DCF-DA fluorescence, when compared to the control group. Selol cotreatment decreased ROS level starting from the 4th hour of incubation (Fig. 2a). Similar results were obtained in cell-free system, Selol decreased fluorescence intensity induced by SNP (Fig. 2b). As glutathione is the first line of defense against oxidative stress, we investigated the effect of Selol on the total intracellular level of glutathione as well as on its reduced (GSH) and oxidized (GSSG) forms. Although the total glutathione level did not significantly change in SNP-treated group (Fig. 3a), a decrease in GSH and an increase in GSSG level were observed (Fig. 3b). Selol treatment reversed glutathione oxidation induced by SNP, resulting in elevation of total (Fig. 3a) and reduced glutathione as well as the decrease of GSSG (Fig. 3b), thus maintaining GSH/GSSG ratio (Fig. 3c). SNP also significantly disturbed the function of antioxidative enzymes. The activity and expression of Se-GPx were decreased by about 43 and 66 %, respectively, an effect completely prevented by Selol treatment (Fig. 4a, b). GR was inhibited by 34 % by SNP (Fig. 4c) with a concomitant increase in the mRNA level for this protein by 138 % (Fig. 4d). Selol treatment prevented the inhibition of GR activity evoked by SNP (Fig. 4c) and significantly reduced the upregulation of its mRNA (Fig. 4d). SNP also inhibited the Se-dependent thioredoxin reductase (TrxR) activity by about 61 % (Fig. 4e) and enhanced its expression by 220 % (Fig. 4f); Selol significantly prevented these effects (Fig. 4e, f). Selol alone, however, moderately increased mRNA for *Txnrd1* (by 53 %, Fig. 4f). To further investigate the cytoprotective action of Selol, we used the Se-GPx inhibitor sodium aurothimalate (SAu). Both SAu, (2 μM) and SNP significantly reduced Se-GPx activity by about 60 % (Fig. 5a), but administration of SAu in combination with SNP did not further reduce Se-GPx activity. Although Selol significantly prevented SNP-evoked Se-GPx inhibition, it showed no effect in the presence of SAu or SNP + SAu (Fig. 5a). As indicated in Fig. 5b, both SNP and SAu reduced PC12 cell viability by about 40 % compared to control. Selol was ineffective when these cells were subjected to SAu or SAu + SNP (Fig. 5b).

## Discussion

Oxidative stress is increasingly recognized as an important mechanism of neurodegenerative disorders and thus has become an attractive therapeutic target. PC12 cells serve as a common model for the investigation of neurotoxic effects of stress [38] as well as neuroprotection induced by candidate drugs [39]. Selenium can be either pro- or antioxidant in various circumstances [29, 40] and may thus be either neuroprotective or cytotoxic [41]. In the present study we demonstrate that Selol potently inhibits SNP-induced dopaminergic PC12 cell death. SNP is among the most widely studied NO donors. Moreover, this NO-releasing compound could exert toxic effects as a result of its breakdown to another toxic species, including CN^−^, Fe^2+^, H_2_O_2_, and [Fe(CN_6_)]^4−^ [13]. Treatment with SNP increased total ROS and ONOO^−^ generation in human dopaminergic neuroblastoma cells (SH-SY5Y) [42]. SNP-evoked toxicity mediated by ROS generation was also demonstrated in rat adrenal pheochromocytoma, retinal neuronal (RGC-5), and murine neuroblastoma N1E-115 cells [43–45]. In addition, this compound affected the expression of oxidative stress-related genes [46, 47]. SNP added to the cell culture could in part mimic the oxidative stress documented in the brains of patients with neurodegenerative disorders. We observed massive cell death with characteristic features of apoptosis in SNP-treated cells.

Potential therapeutic approaches for the treatment of neurodegenerative disorders include Se-based interventions through incorporation of selenoamino acids into antioxidative enzymes [48]. We provided experimental support for the hypothesis that Selol played a vital role in the antioxidative response against SNP treatment, even if coadministered at the time of cytotoxic insult. Sarker et al. have shown that 12 h of pretreatment with Ebselen [2-phenyl-1, 2-benzisoselenazol-3 (2H)-one], a Se-containing heterocyclic organic compound, as well as with an inorganic compound sodium selenite reduced PC12 cell death evoked by SNP [49]. However, in our study, sodium selenite added to the cell culture at the same time with stressor failed to protect from SNP-mediated cell death. It is possible that the ineffectiveness of inorganic selenium donor might be due to lower cell membrane permeability (leading to high toxicity). Moreover, the choice of organic Se compounds over their inorganic counterparts usually stems from their more efficient digestive tract absorption [50, 51]; it is possible that their action also differs significantly at the cellular level. To investigate if Selol could directly influence the levels of free radicals produced by SNP, we performed DCF measurements of ROS in a cell-free system containing SNP and Selol. Our results show that Selol significantly reduced free radical levels, thus suggesting that Selol’s action in the biological material might be also at least partially mediated by its direct influence on SNP. The cytotoxic action of SNP was accompanied by a collapse in the ratio of reduced to oxidized glutathione (GSH/GSSG) [42, 52]. GSH is a cosubstrate of GPx that is responsible for the reduction of organic and inorganic hydroperoxides to water or alcohols with GSSG being a by-product. GSH can be restored either by GR in a NADPH-dependent reaction or by the thioredoxin reductase/thioredoxin couple-TrxR/Trx (rather slow) [53]. Changes in the GSH content mirror closely both oxidative stress and many kinds of pathologies linked to the dysregulation of the antioxidant network. We observed that the GSSG elevation indicated a shift toward more prooxidative cellular milieu and this might be due to direct reaction of GSH with ROS/RNS particularly hydroxyl radical (^•^OH), (NO) (after oxidation to the NO^+^ form) and peroxynitrite (ONOO^−^). The reaction results the formation of thiyl radicals (GS^•^) that are, in turn, combining with other thiyl radicals to form glutathione disulfide [54]. We showed that the protective mechanism of Selol against SNP cytotoxicity included significant inhibition of ROS production along with the restoration of GSH homeostasis, and the expression/activity of antioxidative enzymes. Similarly, Aykin-Burns et al. showed that selenocystine (SeCys) also ameliorated lead-induced imbalance of the GSH/GSSG ratio [55]. In addition, pretreatment with SeMet for 16 h has been shown to protect primary rat hippocampal neurons against the toxicity of ROS generated by iron/hydrogen peroxide (Fe^2+^/H_2_O_2_) or amyloid β. The effect was mediated by increased GPx protein and activity [56]. In our study, the administration of SNP in PC12 cells led to significant depletion of antioxidant activities, which is in accordance with the report of Pandareesh and Anand [52]. However, it could be debated whether the reduction in Se-GPx activity is due to some regulatory events, or might stem from a decline in its expression while GR and TrxR appear to undergo inhibition. Selol significantly ameliorated the SNP-induced inhibition of GSH-dependent antioxidative enzymes (GPx, GR) and TrxR. Similar effect was proved by Song et al.; they showed that mycotoxin-mediated inhibition of GPx and GR was relieved by supplementation of organic or inorganic Se [57]. Moreover, the organic Se–Met (and the inorganic compounds to a lesser degree) increased the expression of mRNAs coding *GPx1* and *GPx4* [57]. The expression of several crucial antioxidative enzymes including *Txnrd1, GPx1*, and *Gsr* is under the control of Nrf2 (nuclear factor-erythroid 2-related factor 2). Nrf2 promotes the regulation of the intracellular redox environment and cytoprotection via binding to the promoter sequences termed antioxidant responsive element (ARE) and up-regulating the transcription of ARE-containing antioxidant genes [58–60]. Treatment of cells with Selol followed by SNP exposure resulted in the increase of target genes expression, thus it is likely that Nrf2 mediates the observed effects. It corresponds with a report where selenite induced protective changes in mitochondrial biogenesis by increasing the level of Nrf1 and nuclear accumulation of Nrf2 [61].

In our study, SNP significantly decreased *GPx1* mRNA expression and in parallel increased *Gsr* and *Txnrd1* mRNAs. We interpreted that the depletion of enzymatic activity of GR and TrxR after SNP exposure led to compensatory upregulation of their expression to cope with the stress. However, poor correlation between GPx activity and its mRNA expression in the presence of SNP may indicate that translational or posttranslational mechanism(s) might be involved in its regulation. Such post-translational modifications might involve not only enzymatic reactions, but also direct free radical-induced damage, such as S-nitrosylation. Such modulation occurs *e.g*. in the case of the thioredoxin—TrxR system [62]. Interestingly, *GPx1* mRNA may be the target of two stop codon-mediated modulation mechanisms: mRNA abundance control (probably via degradation) and a translational change (the meaning of UGA is modified from ‘stop’ to ‘selenocysteine’). Under standard conditions, the UGA stop codon is bypassed and selenocysteine is incorporated, but at conditions of selenium deficiency, this UGA sequence seems to revert to its standard meaning as a termination codon. This additionally leads to a reduction in *GPx1* mRNA, possibly through a mechanism that ensures the removal of aberrant mRNAs that prematurely terminate transcription [63–67]. Together with our observations, this might suggest that under SNP conditions, the supply of selenium becomes a limiting factor, either via Se incorporation, or through Selol’s antioxidative effects (ROS scavenging, induction of gene activities). Therefore, Selol treatment increased the transcription levels of the *GPx1* under condition of SNP that may facilitate the protein synthesis, and may further elevate its activity. Selol at the same time decreased the level of *Gsr* and *Txnrd1* in SNP-treated cells. To further characterize the activation of antioxidant enzymes by Selol under condition of SNP, we used GPx inhibitor (sodium aurothiomalate; SAu). Either SAu or SNP significantly reduced Se-GPx activity when administered alone, but SAu in combination with SNP did not further reduce Se-GPx activity. Selol prevented Se-GPx inhibition caused by SNP, but not by SAu or SNP in combination with SAu. This suggests that the effect of Selol might be linked to the antagonistic action against SNP/SNP-produced ROS, in accordance with our in vitro DCF data. However, blockage of Se access to the enzyme’s catalytic center by SAu could not be ruled out. Our results showed that treatment with SAu prevented the protective effect of Selol against SNP-induced apoptosis, again suggesting the involvement of Se-GPx in the protective effect of Selol against SNP-evoked toxicity. As SAu is also able to inhibit the activity of TrxR [68], this enzyme’s role as a target in Selol-mediated protection cannot be excluded, although its changes in response to Selol treatment are less evident.

In summary, we propose a model whereby Selol modulates the impact of SNP on GPx and other antioxidant enzymes and prevents SNP-induced cell death. This successful in vitro application in a model of ROS-/RNS-mediated cytotoxicity suggests Selol as a promising compound in therapy of diseases related to oxidative/nitrosative damage and dopaminergic cells death. This potentially attractive mechanism deserves further in vivo clarification including in-depth analysis of Selol’s impact on other aspects of neuronal death mechanisms.
